# ProB-Site: Protein Binding Site Prediction Using Local Features

**DOI:** 10.3390/cells11132117

**Published:** 2022-07-05

**Authors:** Sharzil Haris Khan, Hilal Tayara, Kil To Chong

**Affiliations:** 1 Department of Electronics and Information Engineering, Jeonbuk National University, Jeonju 54896, Korea; shazilharis1994@jbnu.ac.kr; 2 School of International Engineering and Science, Jeonbuk National University, Jeonju 54896, Korea; 3 Advances Electronics and Information Research Center, Jeonbuk National University, Jeonju 54896, Korea

**Keywords:** deep neural networks, evolutionary information, local features, machine learning, protein binding sites, structural information

## Abstract

Protein–protein interactions (PPIs) are responsible for various essential biological processes. This information can help develop a new drug against diseases. Various experimental methods have been employed for this purpose; however, their application is limited by their cost and time consumption. Alternatively, computational methods are considered viable means to achieve this crucial task. Various techniques have been explored in the literature using the sequential information of amino acids in a protein sequence, including machine learning and deep learning techniques. The current efficiency of interaction-site prediction still has growth potential. Hence, a deep neural network-based model, ProB-site, is proposed. ProB-site utilizes sequential information of a protein to predict its binding sites. The proposed model uses evolutionary information and predicted structural information extracted from sequential information of proteins, generating three unique feature sets for every amino acid in a protein sequence. Then, these feature sets are fed to their respective sub-CNN architecture to acquire complex features. Finally, the acquired features are concatenated and classified using fully connected layers. This methodology performed better than state-of-the-art techniques because of the selection of the best features and contemplation of local information of each amino acid.

## 1. Introduction

Proteins are large biomolecules crucial for execution of various biological processes, such as catalysing or de-catalysing a metabolic process, DNA replication, creation of antibodies, and transportation of nutrient in the cells of an organism. For these purposes, they need to interact with other biological macromolecules [1]. One such interaction is protein–protein interaction (PPI), which entails the physical interaction of multiple proteins. PPI is crucial for carrying out the biological role of proteins [2,3]. Proteins interact with other proteins using specific binding sites; therefore, identification of these binding sites reveal how a protein performs its biological functions [4,5]. This new insight could potentially aid in the formulation of novel antibacterial drugs [6]. Conventionally, PPIs have been identified using experimental methods such as affinity purification coupled with mass spectrometry and two-hybrid screening [7,8,9]. However, these experimental methods are limited by their high cost and time consumption. Therefore, there is a need for an accurate computational tool to perform the vital task of protein binding sites identification, which will further help in understanding PPIs. Previously, artificial intelligence has attained great attention due to its achievements in solving different biological tasks in genomics and proteomics [10,11,12,13,14].

Various computational techniques have been suggested to identify PPI sites. These methods can be categorized into three domains. Protein structure-based, protein sequence-based, and protein–protein docking based methods. Both protein structure-based and protein docking methods require structural information about proteins, which is usually not available for most proteins; however, sequence information is available for most proteins [15]. In addition, as there is an enhancement in high-throughput screening techniques, a large number of protein sequences can be secured, which has shifted the focus of research towards sequence information based methods. Various computation-based methods employed machine learning techniques, including shallow neural networks [16,17,18,19], random forest [20,21,22], support vector machine [23,24,25], Naïve Bayes [26], conditional random field [27], and ensemble learning [28]. These studies utilized various features extracted from the protein sequences. Evolutionary information [29,30,31] and secondary structures [32,33] are the commonly used features for PPI identification. However, some other features, such as physiochemical, statistical, and biophysical features, which include accessible surface area [22,34], backbone flexibility [35], sequence specificity [36], and protein size [37], are used to predict binding sites.

One effective method is to extract contextual local features of each amino acid in a protein sequence. Thus, various computational techniques employed sliding-window-based techniques to utilize neighboring amino acid sequences to extract features. This sliding-window technique provides flexibility in its application, as it is used in extraction of local information around targeted amino acids [21,22,38]; hence, it is also applied in different problems involving proteins. Examples of such problems include protein structure prediction and protein disorder prediction [39]. In contrast to local features, another method used in the literature is global sequence features to identify interfacial amino acids [23]. Global features can also be used to enhance machine-learning algorithms for predicting binding sites [40,41]. In the past decade, graph convolutional networks have been popularly used in protein-related tasks, such as genomic analysis [42], protein solubility prediction [43], and drug discovery [44]. However, graphs have had little success in solving problems, mainly due to the lack of extraction of higher-order features and the adaption of shallow architectures that caused low performance. In addition, the application of multiple layers and injecting non-linearity led to over-smoothing of the representation of nodes, causing the nodes to converge to a certain value, which leads to reduced performance [45]. Research has shown that over-smoothing problem can be tackled using identity mapping [46]. However, the unavailability of the structural information of majority of the proteins in databases is the main reason for the limited application of graph-based approaches.

Feature selection is of key importance for the identification of binding sites in proteins using sequential information. Each amino acid is unique, and each protein consists of a diverse sequence of amino acids. Therefore, such features are required to represent the effects of entire sequence on each position of the sequence. Evolutionary features were therefore chosen to be the focus of this study because they meet the criteria and have been successful in solving sequence problems in the past [47]. In addition, secondary structure features that best describe the impact of individual amino acids on the whole sequence were used in this study. To utilize the local features, the sliding window approach was used on the extracted features. Fully connected convolutional layers were then used for the extraction of higher-order features, and classification was performed using fully connected neural networks.

## 2. Materials and Methods

### 2.1. Datasets

In this study, we incorporated three benchmark datasets: Dset_72, Dse_186 presented by Murakami and Mizuguchi [48], and PDBset_164 proposed by Singh et al. [49]. Dset_72, PDBset_164, and Dset_186 were constructed using the PDB database, and contained 72, 164, and 186 protein sequences, respectively. All datasets had similar sequence homology of <25%, with a resolution of <3.0 Å. These were annotated datasets; hence, there were a total of 422 unique annotated protein sequences. BLASTClust [50] was used to discard redundant proteins containing 25% sequence similarities with 90% overlapping criteria for any sequence. By discarding the redundant proteins, 395 protein chains remained. These three datasets were merged to form one dataset so that the interacting and non-interacting binding site ratios became similar while dividing it into training and testing sets. A total of 335 protein sequences were extracted as the training set for the model, whereas the remaining 60 protein sequences were used for independent testing. To label a site as a binding site, the amino acid affinity for absolute solvent accessibility had to be <1 Å^2^, both before and after the binding state of protein binding; otherwise, it was labeled as a non-binding site. Available interaction and non-interaction sites were calculated; interaction sites in Dset_72, PDBset_164, and Dset_186 were 1923, 6096, and 5517, respectively, while the non-interaction sites were 16,217, 27,585, and 30,702, respectively. The training set is referred to as train_335, and the test set is referred to as test_60 in this study.

Another dataset provided by Yuan et al. [47] was used for the independent testing of the model. This model contains 315 protein sequences, which are recently solved protein complexes in PDB (2014–2021). Similar criteria for removing redundant proteins were applied to the dataset. This dataset is referred to as test_315 in this study.

### 2.2. Protein Features

Appropriate feature representation is a necessary step in a deep learning framework. In this study, amino acid structural information and evolutionary information for identifying the individual encoding of all amino acids in a protein sequence were used. For evolutionary information, two techniques have been employed: a position-specific scoring matrix and hidden Markov models. The extracted features are described in the following sections.

#### 2.2.1. Structural Information

Structural information on an amino acid is a key feature in the prediction of binding sites in a protein sequence. This feature was extracted by running the DSSP program (v3.1.4, Heidelberg, Germany) [51], which utilizes the information of atoms and bonds between the atoms of an amino acid to predict its secondary structure. These secondary structures were divided into eight categories: α-helix, 310-helix, π-helix, β-turn, β-bridge, β-strand, loop, and bend. In addition, DSSP may not give secondary structures of a few amino acids; hence, a 9-dimensional vector was created with these features, where each dimension represents the secondary structure possessed by an amino acid. This vector was created by adapting the one-hot encoding scheme (possessing one state at a time), where the 9th dimension represents the absence of a secondary structure state. Other structural properties were also calculated by DSSP, which include peptide backbone torsion angles that are represented by 4-dimensional vector with the help of sine and cosine transformations. Furthermore, the solvent-accessible surface area was also extracted and normalized by the possible maximum surface area of the amino acid to provide a relative accessible surface area. Structural features were generated by concatenating these extracted features to get a 14-dimensional vector represented by DSSP features.

#### 2.2.2. Evolutionary Features

Motifs of evolutionarily conserved protein sequences may provide crucial information related to protein binding behavior. Various statistical features can be extracted using this information. These features include the probability of the presence of certain amino acids at specific positions in the sequence to find similar protein sequences. The hidden Markov model (HMM) profile and position-specific scoring matrix (PSSM) were used to extract these features. HMM features were acquired using the HHblits v3.0.3 (Munich, Germany) [52] tool, which utilizes the UniClust30 database (v30, Göttingen, Germany) [53] to align the sequence being analysed. PSSM features were extracted using the PSI-BLAST v2.10.1 (USA) [54], which utilizes the UniRef90 database (v90, Washington DC, USA) [55]. The configurations of both tools were set as suggested by Zeng et al. [56]. Both techniques provided 20 feature vectors as outputs. This output was then normalized between 0 and 1 by taking the maximum and minimum values from the overall features present in the training set for its respective feature type.

### 2.3. Proposed Model

**Figure 1:**
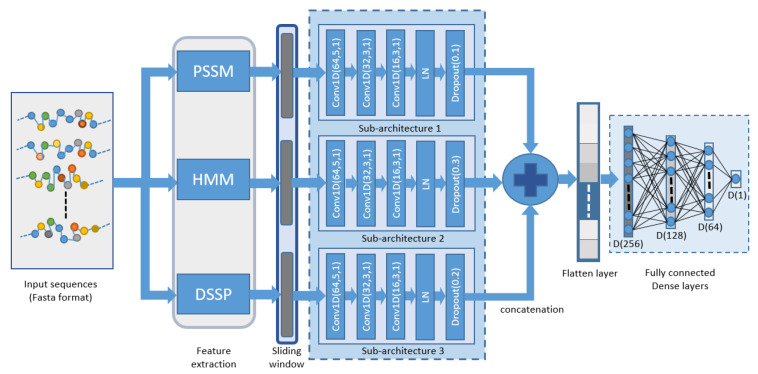
Flow diagram of the proposed model.

The ProB-site is a CNN-based architecture that has been implemented for the prediction of binding sites in a protein sequence, which utilizes the structural information and evolutionary information of the local region of a sequence to predict the state of each amino acid, as shown in Figure 1. Input protein sequences are acquired using PDB database [57], then they are parsed to acquire the desired chain in the FASTA format file. This FASTA file contains the protein sequence, which is fed to the feature extraction block. From the feature extraction block, three types of features are acquired, HMM, PSSM, and DSSP features. The local features of each amino acid were extracted using the sliding window technique upon protein sequence for each feature set separately. Using this process, each amino acid utilizes the information of its neighboring amino acids. The window size was chosen to be odd (2a + 1), where the concerned amino acid is in the middle of the window, and 2a is the number of neighbors around the amino acid. Using a sliding window, we obtained the m × n vector, where m is the vector size, and n is the window size. The value of m is 20, 20, and 14 for the PSSM, HMM, and DSSP features, respectively, and n was selected as 19. These extracted local features were fed to their respective CNN sub-architectures, as discussed below.

This study proposed a CNN Based tool for the identification of binding and non-binding sites in a protein sequence. Three small CNN architectures were utilized for higher feature extraction, and these architectures collectively comprise the complete architecture. Three feature sets were used in this study: each feature set was fed to its respective CNN module to generate higher features for classification.

CNN sub-architecture 1 is a three-layered convolutional model with three convolutional layers connected in succession. The filter sizes (kernel size) of the convolutional layers were 5, 3, and 3 with 64, 32, and 16 filters, respectively. The stride length was 1, and the layers were activated using the ReLU function. The output of the third convolutional layer was fed to layer normalization. After normalization, a dropout layer was applied to prevent overfitting. CNN sub-architectures 2 and 3 were created in parallel with the same configurations as architecture 1. However, the dropout ratio in the dropout layer in the three parallel sub-architectures was different from architectures 1 to 3 with dropout ratios of 0.1, 0.3, and 0.2, respectively. The PSSM, DSSP, and HMM features were fed to their respective CNN architectures from 1 to 3. The extracted higher-dimensional features acquired from each sub-architecture were then concatenated. These features were then passed to dense layers for classification after the application of the flattened layer. Fully connected dense layers were used to classify the binding sites in the protein sequences. The consecutive dense layers consisted of 256, 128, 64, and 1 neuron, respectively, with the first three layers activated by the ReLU function [58] and the last layer activated by the sigmoid function [59] to acquire the binary output. The mathematical representation of both the activation functions ReLU and sigmoid are given in Equations (1) and (2), respectively.

(1) RELU(f)=maximum(0,f)

(2) $$Sigmoid\left(f\right)=\frac{1}{1+\exp(-f)}$$

This study implemented a stochastic gradient descent optimizer to optimally find the parameters and implemented binary cross-entropy as a loss function [60]. The mathematical expression of binary cross-entropy is given in Equation (3), where n represents the number of samples, $a_{i}$ represents the actual label, and $b_{i}$ represents the output of the proposed model.

(3) $$BCE=-\frac{1}{n}\sum_{i=1}^{n}\left(a_{i}.\log b_{i}+\left(1-a_{i}\right).\log\left(1-b_{i}\right)\right)$$

In this study, a hyperparameter tuning approach was used to determine the optimum values of the various parameters in the architecture. The range of values of the hyper-parameters is given in Table 1. The subsequent convolution layers were smaller than the preceding layers. In addition, the sizes of the corresponding convolutional layers among all sub-architectures were kept the same to acquire feature sets with suitable dimensions for concatenation and to avoid any mismatch (e.g., the first convolutional layer of the first, second, and third sub-architectures had the same parameters).

## 3. Results and Discussion

### 3.1. Evaluation Metrics

For PPI binding and non-binding site prediction, binding and non-binding sites were represented by positive and negative samples, respectively. For the performance evaluation of our model and the previous model, we utilized six evaluation metrics of the measurements. Accuracy (ACC), recall, precision, F-measure (F1), Matthews correlation coefficient (MCC), area under the ROC curve (AUROC), and area under the precious-recall curve (AUPRC). The equations for the selected metrics are as follows.

$$ACC=\frac{TP+TN}{TP+TN+FP+FN}$$

$$Recall=\frac{TP}{TP+FN}$$

$$Precision=\frac{TP}{TP+FP}$$

$$F1=2\times \frac{TP}{2TP+FP+FN}$$

$$MCC=\frac{\left(TP\times TN)-(FP\times FN\right)}{\sqrt{\left(TP+FP)\times (TP+FN)\times (TN+FP)\times (TN+FN\right)}}$$

where TP is the true positive and TN is the true negative, representing the correctly predicted binding and non-binding sites, respectively. Furthermore, FP is a false positive and FN is a false negative, representing the false prediction of binding and non-binding sites, respectively.

### 3.2. Window Size Selection

**Figure 2:**
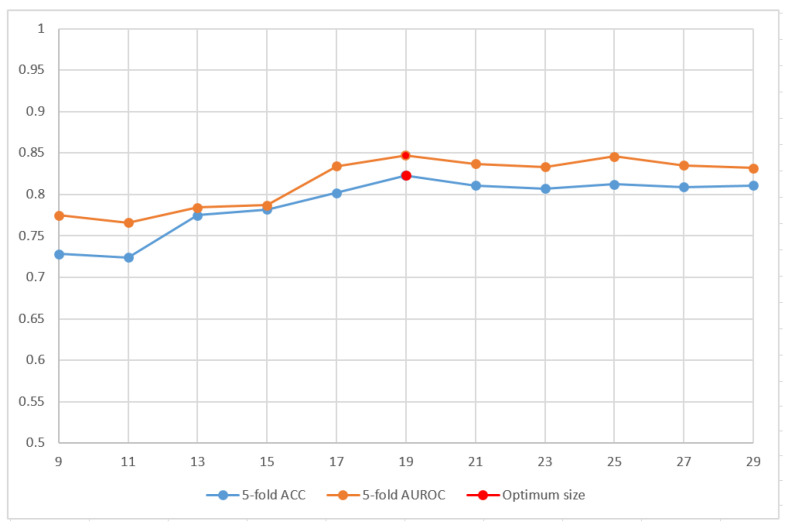
Performance analysis of the model using multiple window size through 5-fold cross-validation.

The proposed model was trained using a 5-fold cross-validation of the training dataset, where the dataset was randomly divided into five folds. By implementing five-folds, each time, the model was trained four times and tested on the remaining fold. This process was repeated five times for each fold, and the results were averaged as the overall validation performance. For various window sizes, 2n + 1 with a value of n ranging from 4 to 14, the 5-fold cross-validation model was applied to determine the optimum window size for the research problem. The results of this procedure are shown in Figure 2. As the size of the window increases, the computational complexity of the model also increases. Hence, after judging the pattern of accuracy and AUROC from the graph, a window size of 19 was selected because it yielded the best results for 5-fold cross-validation; furthermore, as the window size increased, there was no visible improvement in the performance of the model, and there was an added expense of computational complexity.

### 3.3. Evaluation of Features

To understand individual contribution of evolutionary and Secondary structures information; the proposed model is trained and test using evolutionary (PSSM and HMM) and DSSP features separately. In addition, the proposed model is trained and tested by using all three features. Results of these three settings are shown in Table 2. A test dataset of 60 protein sequences was used. Secondary information has proved to be more useful in the training of the given model, and it outperformed the evolutionary features in all aspects, as shown in Table 2. The secondary structure contains amino acid structural information that is utilized by the model more efficiently than evolutionary information. Both feature types contributed to the enhancement of the overall architecture.

### 3.4. Performance Comparison with Other Techniques

The results of this study were compared with five sequence-based (PSIVER [48], SCRIBER [61], DELPHI [62], ProNA2020 [63], and DLPRED [64]) and four structure-based (SPPIDER [19], DeepPPISP [56], MaSIF-site [65], and GraphPPIS [47]) binding site classifiers. The performance of the state-of-the-art architecture was evaluated for an individual test set of 60 amino acids. The results of relevant previous studies were acquired from Yuan et al. [47]. The proposed architecture utilizes sequential information, as the structural information of the majority of proteins is not available in online databases. Additionally, the structural information of proteins is complex; hence, creating an optimal model and training is not an easy task. The proposed architecture has implemented the amino acid structural information along with the evolutionary information, which gives it an edge over other sequential-based techniques, as shown by the performance in Table 3. In this study, local information for each amino acid was considered for training and prediction using the windowing technique, adding additional information while predicting each amino acid. This approach has led to better performance than state-of-the-art techniques. The ProB-site achieved an accuracy of 0.799, precision of 0.407, and MCC of 0.428, which is an improvement over the results of previous models. To further test the generality of the ProB site, the trained model was tested on the test_315 dataset and compared with the results of a state-of-the-art model for the same dataset. The results for previous models were obtained from Yuan et al. [47]. The results in Table 4 demonstrate that ProB-site outperformed the previous techniques for the test_315 dataset.

## 4. Webserver

The proposed model has been provided as a webserver tool to the research community. ProB-site is made freely available at http://nsclbio.jbnu.ac.kr/tools/ProB-site/, accessed on 27 June 2022. It is a user-friendly tool. The user needs to provide a PDB ID and Chain identifier as input. Since the computation of evolutionary features takes time, the features of protein sequences used in this research were pre-stored to speed up their predictions. The output of this tool is the protein sequence with the predicted binding site. Furthermore, the source code, pre-trained model, and all necessary information to replicate the proposed model are made available at Github: (https://github.com/sharzil1994/ProB-site, accessed on 20 June 2022).

## 5. Conclusions

For protein binding site prediction, it is necessary to select optimum features that can represent the properties of individual amino acids, as well as the properties that represent the impact of the whole sequence on an individual amino acid. This study considered both requirements and used evolutionary and secondary structure information prediction. Since the majority of proteins in the databases do not have protein structures available, the requirement of a sequence-based approach is necessary to find binding sites for proteins; this issue has been addressed using this approach. A deep neural network architecture has been proposed through ProB-site, which has utilized sub-architectural convolution networks to extract higher-order features and used deep neural networks for the prediction of binding sites of the protein sequences, thereby achieving improved performance as compared to the previous techniques. The ProB-site achieved an accuracy of 0.799 and ARROC of 0.844 on the benchmark dataset. This model was tested on a new dataset of test_315, which yielded promising results, with an MCC of 0.351 and AUPRC of 0.446.

## Figures and Tables

**Table 1:** Parameter ranges for hyperparameter tuning.

Layers	Parameters
Convolution layer 1	32, 64, 128 (Kernel Size 3, 5, 7)
Convolution layer 2	8, 16, 32, 64 (Kernel Size 3, 5)
Convolution layer 3	8, 16, 32 (Kernel Size 3, 5)
Dropout	0.1, 0.15, 0.2, 0.25, 0.3, 0.35, 0.4
Dense layer 1	256, 128, 64
Dense layer 2	128, 64, 32
Dense layer 3	64, 32, 16, 8

**Table 2:** Comparison for the individual effect of features on the proposed model (ProB-site).

Feature Set	ACC	Precision	Recall	F1	MCC	AUROC	AUPRC
Evolutionary features	0.651	0.224	0.675	0.311	0.149	0.651	0.243
(PSSM + HMM)							
Secondary structures	0. 752	0. 354	0.755	0. 475	0.380	0.824	0.428
(DSSP)							
All Features	0.799	0.407	0.612	0.517	0.368	0.844	0.467
(PSSM + HMM + DSSP)							

**Table 3:** Proposed model (ProB-site) compared with the previous techniques in the literature using Test_60 data set.

Method	ACC	Precision	Recall	F1	MCC	AUROC	AUPRC
PSIVER	0.561	0.188	0.534	0.278	0.074	0.573	0.190
SCRIBER	0.667	0.253	0.568	0.350	0.193	0.665	0.278
DELPHI	0.697	0.276	0.568	0.372	0.225	0.699	0.319
ProNA2020	0.738	0.275	0.402	0.326	0.176	N/A	N/A
DLPred	0.682	0.264	0.565	0.360	0.208	0.677	0.294
SPPIDER	0.752	0.331	0.557	0.415	0.285	0.755	0.373
DeepPPISP	0.657	0.243	0.539	0.335	0.167	0.653	0.276
MaSIF-site	0.780	0.370	0.561	0.446	0.326	0.775	0.439
GraphPPIS	0.776	0.368	0.584	0.451	0.333	0.786	0.429
ProB-site	0.799	0.407	0.612	0.517	0.368	0.844	0.467

**Table 4:** Performance comparison of proposed model (ProB-site) with previous models using test_315 dataset.

	Deep PPISP	SPPIDER	MaSIF-Site	GraphPPIS	ProB-Site
MCC	0.169	0.294	0.304	0.336	0.351
AUPRC	0.256	0.376	0.372	0.423	0.446

## Data Availability

Online tool is avaliable at (http://nsclbio.jbnu.ac.kr/tools/ProB-site/, accessed on 27 June 2022) and data and traied model used in this research are avaliable at (https://github.com/sharzil1994/ProB-site, accessed on 27 June 2022).
